# Reply to Kocsmár, É.; Lotz, G. Comment on “Skrebinska et al. Who Could Be Blamed in the Case of Discrepant Histology and Serology Results for *Helicobacter pylori* Detection? *Diagnostics* 2022, *12*, 133”

**DOI:** 10.3390/diagnostics13132273

**Published:** 2023-07-05

**Authors:** Ilva Daugule, Francis Megraud, Marcis Leja

**Affiliations:** 1 Institute of Clinical and Preventive Medicine, Faculty of Medicine, University of Latvia, LV-1586 Riga, Latvia; marcis.leja@lu.lv; 2 French National Reference Centre for Campylobacters and Helicobacters, Bacteriology Laboratory, Bordeaux University Hospital, 33000 Bordeaux, France; francis.megraud@u-bordeaux.fr; 3 INSERM U1053 BaRITOn, University of Bordeaux, 33000 Bordeaux, France; 4 Digestive Diseases Centre “GASTRO”, LV-1079 Riga, Latvia

Dr. Kocsmár and Dr. Lotz have made important comments [1] and raised good questions about recommendations for the broader use of immunohistochemistry (IHC) as well as polymerase chain reaction (PCR) testing in cases where an etiological role of *H. pylori* is clinically suggested, but histopathological confirmation of *H. pylori* is not possible.

Indeed, the use of IHC could be more valuable in Giemsa-negative cases without inflammatory activity in which the etiological role of *H. pylori* is suggested by clinical, anamnestic or other data. 

Although this leads to the idea of the routine use of IHC as the primary staining method, instead of Giemsa, its rather higher costs of analysis should be taken into account.

On the other hand, PCR testing could also be an option in cases where an etiological role of *H. pylori* is suspected. We (could) also support the idea of the use of PCR in doubtful cases. Moreover, it turns out that the broader use of molecular methods is also recommended by the last edition of the Maastricht guidelines [2].

In summary, the topic of routine use of IHC and PCR for *H. pylori* identification should be included in expert discussions as well as in the preparation process of the next guidelines for the management of *H. pylori* infections.
